# Highly Efficient Transgenesis in Ferrets Using CRISPR/Cas9-Mediated Homology-Independent Insertion at the *ROSA26* Locus

**DOI:** 10.1038/s41598-018-37192-4

**Published:** 2019-02-13

**Authors:** Miao Yu, Xingshen Sun, Scott R. Tyler, Bo Liang, Anthony M. Swatek, Thomas J. Lynch, Nan He, Feng Yuan, Zehua Feng, Pavana G. Rotti, Soon H. Choi, Weam Shahin, Xiaoming Liu, Ziying Yan, John F. Engelhardt

**Affiliations:** 1 Department of Anatomy and Cell Biology, Carver College of Medicine, University of Iowa, Iowa City, IA 52242 USA; 2 College of Life Science, Ningxia University, Yinchuan, Ningxia 750021 China

## Abstract

The domestic ferret (*Mustela putorius furo*) has proven to be a useful species for modeling human genetic and infectious diseases of the lung and brain. However, biomedical research in ferrets has been hindered by the lack of rapid and cost-effective methods for genome engineering. Here, we utilized CRISPR/Cas9-mediated, homology-independent insertion at the *ROSA26* “safe harbor” locus in ferret zygotes and created transgenic animals expressing a dual-fluorescent Cre-reporter system flanked by PhiC31 and Bxb1 integrase *att*P sites. Out of 151 zygotes injected with circular transgene-containing plasmid and Cas9 protein loaded with the *ROSA26* intron-1 sgRNA, there were 23 births of which 5 had targeted integration events (22% efficiency). The encoded tdTomato transgene was highly expressed in all tissues evaluated. Targeted integration was verified by PCR analyses, Southern blot, and germ-line transmission. Function of the *ROSA26*-CAG-^LoxP^*tdTomato*^StopLoxP^*EGFP* (*ROSA*-TG) Cre-reporter was confirmed in primary cells following Cre expression. The Phi31 and Bxb1 integrase *att*P sites flanking the transgene will also enable rapid directional insertion of any transgene without a size limitation at the *ROSA26* locus. These methods and the model generated will greatly enhance biomedical research involving lineage tracing, the evaluation of stem cell therapy, and transgenesis in ferret models of human disease.

## Introduction

Animal models of human diseases are indispensable for dissecting disease pathophysiology and developing therapies. While mice have been extremely helpful in modeling human diseases, in some cases they are inadequate due to species-specific differences in the cell biology of the affected organ and/or the evolutionary divergence of their genome. The domestic ferret (*Mustela putorius furo*) has proven an excellent species for modeling certain human diseases when mice have failed, and technologies for genetic engineering in ferrets have expanded the potential use of this species for human disease research. However, until now, techniques for generating transgenic and knockout ferrets have predominantly relied upon genetic manipulation of fibroblasts followed by somatic cell nuclear transfer^1,2^. Such an approach is labor intensive and expensive. More recently, both clustered regularly interspaced short palindromic repeat (CRISPR)/Cas9- and transcription activator-like effector nuclease (TALEN)-mediated approaches have been used to generate knockout ferrets^3,4^. Here, we present a rapid method for generating transgenic ferrets using CRISPR/Cas9-mediated targeted insertion at the *ROSA26* locus in ferret zygotes.

The domestic ferret is traditionally known for its ability to model pandemic and seasonal influenza virus infection and transmission to the lung^5^. Additionally, ferrets develop multi-organ lethal disease following Ebolavirus infection^6^ and can replicate and transmit severe acute respiratory syndrome coronavirus^7^. The similar cellular distribution of viral receptors in the lung between humans and ferrets likely accounts for the ability of the ferret to serve as a good model of influenza infection^8,9^. Further, the conserved stem cell biology in the lung between these two species also likely contributes to the ability of ferrets to model bronchiolitis obliterans in an allograft lung transplant model^10,11^. Ferrets have also been used to study aspects of cortical neuronal development and neural progenitor cells^12–14^, and recently, knockout ferrets enabled the discovery an evolutionary mechanism involved in determining cerebral cortical size in humans for which conservation in mice is lacking^4^.

The development of techniques to clone ferrets by somatic cell nuclear transfer^15^ enabled the generation of the first genetically engineered ferret model of cystic fibrosis (CF)^2^, a recessively inherited genetic disease caused by mutations in the gene encoding cystic fibrosis transmembrane conductance regulator (*CFTR*). Unlike mice, *CFTR*-knockout ferrets develop multi-organ disease of the lung, pancreas, gallbladder, and liver^1,3,16–19^, whereas both mouse and ferret models contract different forms of intestinal disease^20^. Pancreatic disease, which lead to diabetes in CF patients, is also well conserved in CF ferrets^21–23^. Thus, the domestic ferret appears to be well positioned to model diseases of the lung and pancreas in situations where mouse models have failed.

The completion of the ferret genome^24^ has aided our understanding of why ferrets model human lung diseases so well. Generally speaking, mice are genetically more distant from humans at the protein level than ferrets^24^. For example, with respect to several classes of proteins potentially important for lung inflammation and remodeling, humans and ferrets exhibit significantly greater protein conservation than that observed between humans and mice (GO Terms: Cytokine Activity (Figure S1A, P = 1.47E-12), Receptor Complex (Figure S1B, P = 9.24E-9), and Extracellular Matrix (Figure S1C, P = 1.05E-13)). In this context, mice lack a direct ortholog of the IL-8 gene, which is present in ferrets and humans, and instead have three IL-8 paralogs (CXCL1/KC, CXCL2/MIP-2, and CXCL5-6/LIX)^25^. Moreover, given the conservation in ferret and human genes related to brain development (Figure S1D, P = 7.85E-9) and visual perception (Figure S1E, P = 8.14E-9), it is not surprising that disruption of the ferret abnormal spindle-like microcephaly-associated (*ASPM*) gene accurately models the most common recessive microcephaly genetic disease^3,4^, and that this species has binocular vision^26^ that closely models multisensory visual processing in humans^27,28^. Notably, both of these attributes are not conserved in mice. Thus, an opportunity exists to create new genetically defined ferret models that share disease-relevant characteristics with humans and better predict the clinical success of therapeutics.

Genetic information provided by the ferret genome prompted us to develop new techniques for rapid genome engineering in ferrets. Recently, the CRISPR/Cas9 system has become a mainstream tool in which to quickly generate transgenic models using zygote injection^29^. CRISPR/Cas9 enables efficient sequence-specific mutations via the homology-directed repair (HDR) and non-homologous end-joining (NHEJ) pathways. Typically, the HDR-mediated gene repair strategy has been used to establish targeted knock-in alleles with donor DNA templates, whereas the NHEJ pathway has been used to generate loss-of-function alleles by frame shift-mediated mutations^30^. NHEJ is the dominant DNA repair pathway in dividing and non-dividing cells, whereas HDR-mediated editing has a comparatively low recombination rate (reported in the range of 0–20%)^31,32^. More recently, NHEJ-mediated targeted insertion in zygotes and embryos has evolved as a method for knock-in transgenesis in *C*. *elegans*^33^, zebrafish^34,35^, and *Xenopus tropicalis*^36^. While CRISPR/Cas9-mediated HDR methods of large transgene-directed insertions have been reported in the literature for mouse zygotes^32^, to our knowledge, NHEJ-mediated targeted insertion in zygotes has yet to be evaluated in mammals.

Here, we report the use of ribonucleoprotein (RNP) Cas9/sgRNA complexes^37^ for ferret zygote microinjections and the targeted insertion of a large transgene into the *ROSA26* locus using a NHEJ knock-in strategy previously established in somatic cells of mice^38^. Using this approach, a Cre-reporter transgenic ferret with a convertible fluorescent reporter system was generated. The Cre-recombinase dual reporter system contains a CAG promoter, membrane-bound *tdTomato* (mT), terminator, and membrane-bound *EGFP* (mG) elements. Additionally, this transgene insertion is flanked by a Dual Integrase Cassette Exchange (DICE) system^39^ to allow for rapid insertion of additional transgenes flanking the Cre-reporter or the directional exchange of the Cre-reporter for another transgene. The DICE system utilizes two phage integrase enzymes that recognize specific DNA sequence motifs within the donor and genomic target locus to facilitate recombinase-mediated cassette exchange. If one recombinase is utilized it will direct insertion only at the DNA recognition motif. This system offers complete control of gene knock-in, including orientation and copy number without a size limitation^39^. This flexible transgenic ferret model will enable highly efficient gene manipulation in ferret zygotes and facilitate both lineage tracing and stem cell therapy studies in ferrets.

## Results

### Strategy for the generation of *ROSA26* knock-in ferrets

The goal of this study was to develop an efficient method for creating a knock-in at the *ROSA26* locus in ferrets, while also generating a generally useful transgenic ferret model for Cre-mediated lineage tracing and stem cell transplantation studies. To identify the ferret *ROSA26* locus, we aligned the ferret shotgun contig library (NCBI, NW_004569143) with the murine *Rosa26* exon 1 sequence and identified the ferret *ROSA26* exon 1 sequence. The ferret *ROSA26* exon 1 and promoter region was compared to those previously identified in the rat^40^ and pig^41^ and we selected a region within the ferret *ROSA26* intron 1 (ENSEMBL genomic location GL896899 32472545–32472606) for targeting of the transgene.

**Figure 1:**
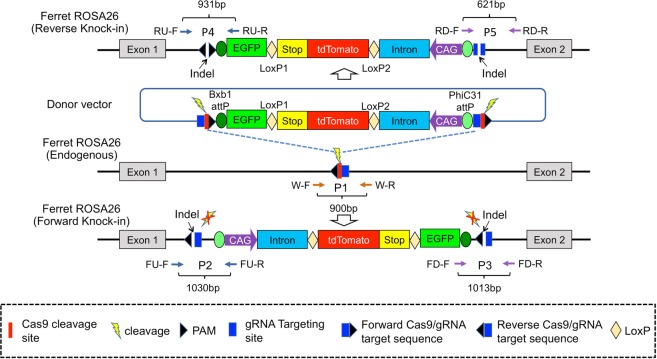
Schematic strategy for targeted integration at the ferret *ROSA26* locus and potential outcomes. Schematic shows the strategy of Cas9-mediated gene targeting and the donor vector. The transgene cassette of the donor plasmid contains a CAG promoter with intron driving expression of the ^LoxP^*tdTomato*^StopLoxP^*EGFP*-PolyA transgene with flanking *Bxb1/PhiC31 attP* integrase sites. The strategy incorporates the Cas9 gRNA sequences targeting the *ROSA26* intron 1 into the flanking sequence of the donor plasmid. After injection of the Cas9 RNP complex into zygote pronuclei, the donor plasmid and chromosome are synchronously cleaved, allowing for efficient integration of the donor DNA. Integration is expected to occur more frequently in the reverse orientation since the gRNA targeting sequence remains intact in the forward insertion if indels are not generated. P1–5 indicate the location of primer pairs for identification the integration junctions and orientations. Unlabeled annotations used in this figure are denoted in the box.

By combining the CRISPR ribonucleoprotein (RNP) technique^37^ with an NHEJ-based insertion strategy for gene editing^38^, we designed a CRISPR/Cas9-mediated, homology-independent, genome-editing strategy that utilized zygote co-injection of a donor plasmid and an RNP complex of Cas9/*ROSA26-*sgRNA (Fig. 1). We first designed sgRNAs targeting intron 1 of the ferret *ROSA26* locus, selecting guides with high *in vitro* cleavage efficiency and low off-target scores (data not shown). These guides cut both the endogenous *ROSA26* genomic locus and both sides of the 8 kb transgene cassette of the donor plasmid (Fig. 1). The transgene cassette contained a CAG promoter with an intron driving expression of a ^LoxP^*tdTomato*^StopLoxP^*EGFP* reporter gene. PhiC31 and a Bxb1 *att*P integrase sites were introduced into the flanking sequences of the reporter transgene for their potential use in DICE-mediated transgenesis^39^.

### CRISPR/Cas9 mediates highly efficient homology-independent targeted insertion at the *ROSA26* locus in ferret zygotes

**Figure 2:**
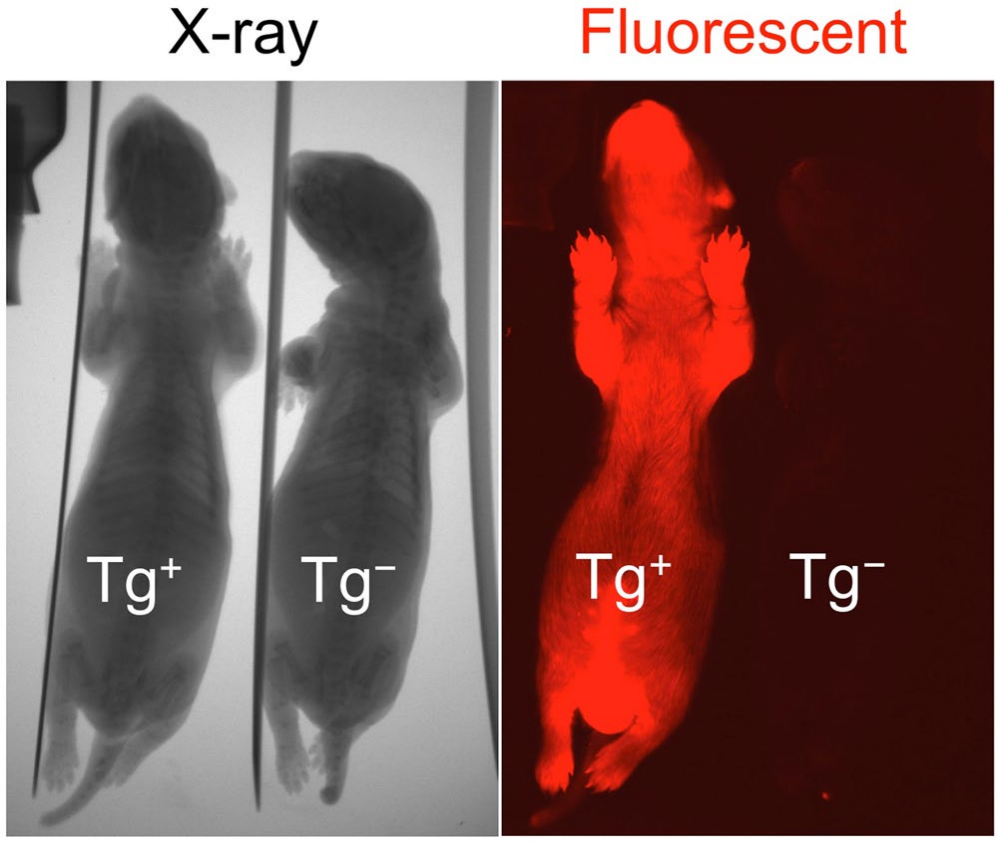
Whole body tdTomato expression in a transgenic ferret. tdTomato transgene expression is shown for a juvenile F1 transgene positive ferret founder A1 (Tg^+^) and transgene negative littermate (Tg^−^). X-ray images are shown in the left panel and fluorescent images are shown in the right panel.

The Cas9 protein from *Streptococcus pyogenes* was pre-complexed with the *ROSA*26-sgRNA to generate the RNP complex. Ferret zygotes were generated from Sable coat-color ferret crosses as previously described^42^ and co-injected with the RNP complex and donor plasmid. This approach of using Cas9/*ROSA*26*-*sgRNA RNP to synchronously cleave the *ROSA26* genomic locus and the donor plasmid DNA led to highly efficient NHEJ-mediated targeted integration of the transgene cassette (Table 1). In total, 151 out of 179 injected zygotes (84%) developed to the 2-cell stage and were transplanted into five pseudopregnant jills (Table 1). Twenty-three kits were born from the these transferred embryos (15.23%), and six of the twenty-three kits (26%) showed expression of the tdTomato transgene, as assessed by an *en face* body scan (Fig. 2). Two of the six transgenic kits (A3 and E1) did not survive (Table 1). However, the survival rate between transgenic kits and non-transgenic kits was not significantly different (data not shown).

**Table 1:** The efficiency and outcome of Cas9-mediated knock-in at the ferret *ROSA26* locus.

Experiment	Zygotes Injected	Zygotes Transferred	Births	tdTomato Positive	ROSA Insertion	Random Insertion	Orientation (F/R)
A	21	18	2	2	2 (A1, A3)	0	F-A1; R-A3
B	37	34	6	2	2 (B2, B5)	2 (B2, B5)	R-B2; F-B5
C	45	39	5	0	—	—	—
D	41	33	4	1	1 (D1)	0	F-D1
E	35	27	6	1	0	1 (E1)	NA
**Total**	**179**	**151**	**23**	**6**	**5**	**3**	**F-3 R-2**

F, forward; NA, unidentified; R, reverse.

**Figure 3:**
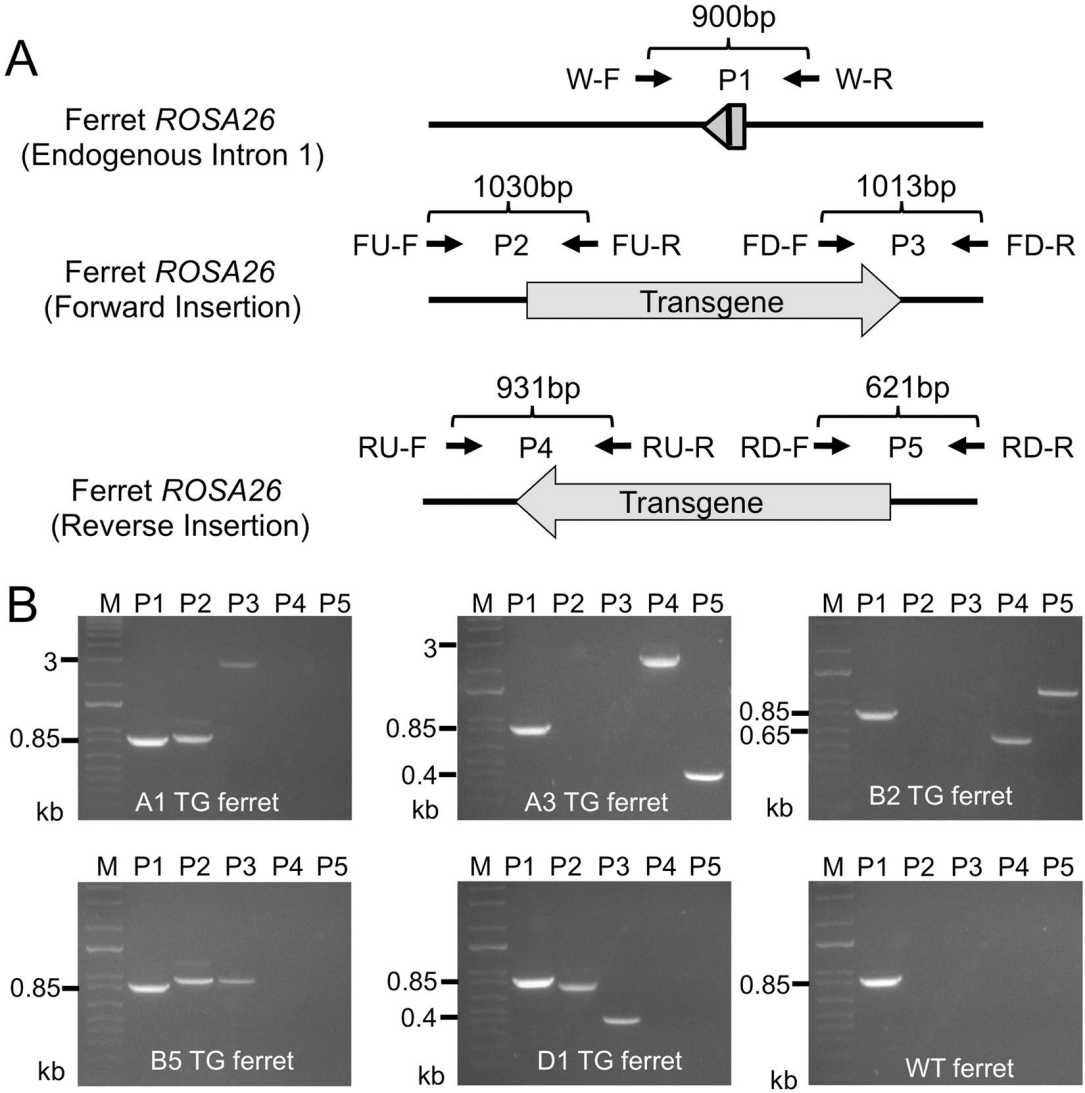
Genotyping of transgene integration and orientation by PCR analysis. To map the orientation of the transgene integration events at the *ROSA26* locus and to characterize indels at the 5′ and 3′ junctions of the insertion site, genomic DNA from F0 founders with observed *en face* tdTomato expression and non-transgenic wild-type ferrets were analyzed for transgene integration using a PCR assay. (**A**) Schematic of the PCR strategy and primers used for mapping the direction of integration events. (**B**) Representative gel images of PCR products (P1-P5) for the indicated transgenic (TG) founder ferrets and a non-transgenic wild-type (WT) animal (see Table 2 for primer descriptions). The predicted sizes of PCR products varied among distinct TG ferret founders due to different sized indels generated from error-prone NHEJ-mediated integration. The sizes of these indels were confirmed by Sanger sequencing of the PCR products (Fig. 4). M, lane denotes 1 kb plus marker.

To confirm NHEJ-mediated targeted insertion and the orientation of the transgene at the *ROSA26* locus, genomic DNA from the four surviving ferrets (A1, B2, B5 and D1) and two non-surviving kits (A3 and E1) were analyzed by PCR using primers that flanked the two potential outcomes of the insertion (forward and reverse) (Fig. 1 and Table 2). Of note, this approach utilized self-inactivating gRNA target sites that theoretically should direct insertion of the transgene cassette in the reverse, but not forward, direction at the *ROSA26* gene. This PCR analysis demonstrated that five (A1, A3, B2, B5 and D1) out of the six tdTomato-expressing transgenic ferret kits had targeted insertions at the *ROSA*26 locus (Fig. 3). However, no PCR product was amplified from genomic DNA of the sixth kit (E1) when using standard primers that flanked the insertion site (data not shown). We hypothesized that a large deletion may have occurred in both flanking region for the E1 insertion and thus walked primers up to 1 kb away from the targeting site and still could not amplify a PCR product. Thus, we assume that the E1 transgenic animal randomly integrated the transgene. Of the five-targeted knock-in kits, three demonstrated a forward orientation of the transgene and two demonstrated a reverse orientation of the transgene at the *ROSA26* locus (Fig. 3, Table 1). Of note, all examined transgenic founders were heterozygotes for the transgene insertion at the *ROSA26* locus, and three of these founders (B2, B5 and E1) contained random transgene integration events of which a subset appeared to be mosaic based on the  intensity of Southern blot banding patterns presented below (Table 1).

**Table 2:** Sequences of primer sets and conditions used for genotyping.

Inserted orientation	Primer ID (F/R)	Primer pair ID	Sequence (5′→3′)	Tm (°C)	Product size (bp)
Wild type	W-F	P1	CCTAACCAAAGGGATGCT	56	900
	W-R		AATGAGCGAAACCACTGAG		
Forward insertion	FU-F	P2	GGAGGAATAAATACATAACTGG	57	1030
	FU-R		ACTGACGGTCGTAAGCAC		
	FD-F	P3	TGTTTCAGTGGGTATGGC	57	1013
	FD-R		GTTTGTTAGGCTGGAAGG		
Reverse insertion	RU-F	P4	CAATCACTCTGCCTTTAACCTC	59	931
	RU-R		TGGGCTCTATGGCTTCTG		
	RD-F	P5	ATGAACTAATGACCCCGTAA	60	621
	RD-R		CCAAATGAGCGAAACCAC		

bp, base pair; FD, forward downstream; FU, forward upstream; RD, reverse downstream; RU, reverse upstream; Tm, annealing temperature; W, wild type.

**Figure 4:**
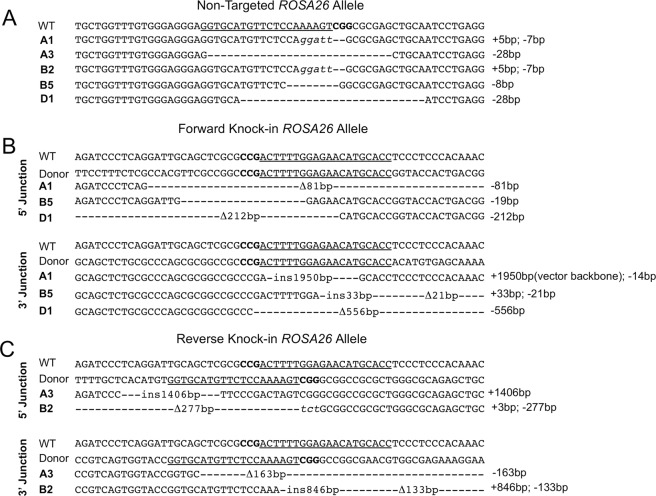
Characterization of indels at the transgene integration sites. The PCR-specific amplicons as shown in Fig. 3 were gel extracted and analyzed by Sanger sequencing. (**A**) Indels created at the non-targeted *ROSA*26 locus for the indicated founder animals. (**B**,**C**) Indels created at the targeted *ROSA*26 locus for (**B**) forward and (**C**) reverse integration events. Wild-type and donor sequences are shown at the top with the targeting gRNA site underlined and protospacer adjacent motif (PAM) sequences bolded. The dash lines denote deletions (Δ) and insertions (ins) with the number of nucleotides indicated. Italics font denotes mutations at the indel site.

To further validate the integrity of the insertion site in the five transgenic animals, the PCR products that were amplified from both sides of the transgene insertion were purified for sequencing analysis. Results from this analysis demonstrated variable-sized indels at the insertion site (Fig. 4). Furthermore, the transgenic founders A1 and A3 contained the transgene together with the plasmid backbone inserted at the *ROSA26* locus. All of the five founders with the targeted transgene integration contained indels of smaller size at the non-targeted *ROSA26* allele. These findings demonstrate that Cas9/sgRNA cleavage at the *ROSA26* locus was highly efficient in the zygotes that contained targeted insertions.

**Figure 5:**
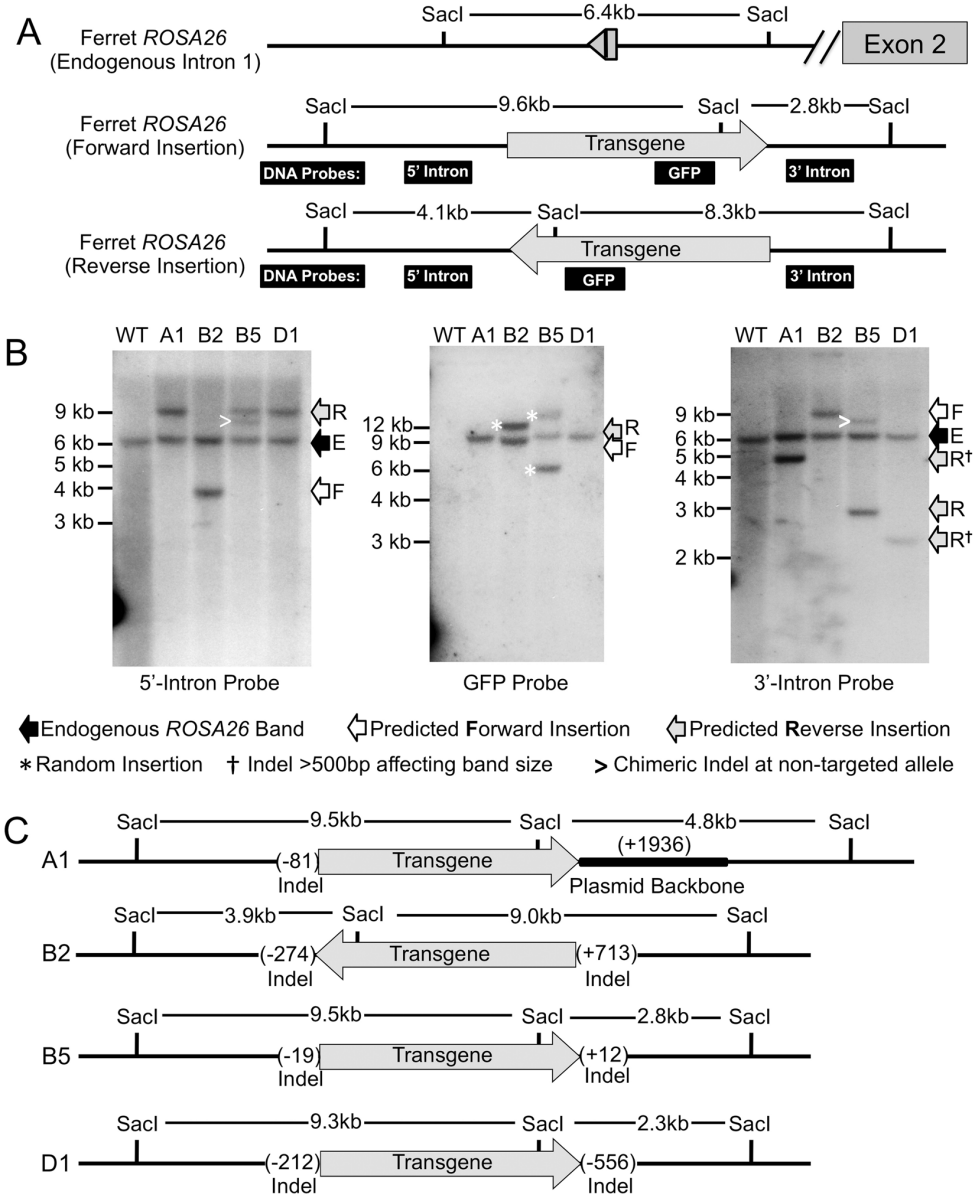
Southern blot analysis of transgenic founder genomic DNA. (**A**) Schematic drawing of hybridization probes for the external arms of 3′-intron and 5′-intron and the *EGFP* transgene, with predicted sizes of Sac1 digested bands for the non-targeted and forward- and reverse-integrated transgenes, respectively. (**B**) Representative Sac1-restricted Southern blots following hybridization with the indicated probes for the four surviving transgenic ferrets. Arrows to the left of each blot denote bands for the endogenous intact locus (E), forward (F)- and reverse (R)-orientated insertion of the transgene cassette. The extra bands seen in B2 and B5 transgenic ferrets with the *EGFP* probe (white asterisks) are suggestive of a random integration event in these animals. Other annotations on the blots are marked in the legend at the bottom. (**C**) Schematic showing the predicted transgene orientation and size of indels at both the 5′ and 3′ junctions of the transgene insertion site for the indicated transgenic animals as determined by results of sequencing and Southern blotting.

Targeting insertions of the transgene were also confirmed by a Southern blotting assay using probes that specifically detected part of the transgene (*EGFP*) and external 3′- and 5′- regions adjacent to the *ROSA*26 targeting site (Fig. 5A). As expected, a 6.4-kb band corresponding to the endogenous *ROSA*26 locus was detected in all ferret genomic DNA samples using intronic probes, confirming the presence of a non-transgenic allele in all examined ferrets (Fig. 5B). Of importance, a specific band with a predicted size of 9.6 kb for a forward insertion of the transgene cassette was observed in A1, B5 and D1 animals using the 5′-intron and the internal *EGFP* probes (Fig. 5B). Equally noteworthy, results of Southern blotting for the A1, B5, D1 transgenic animals also showed different sized unpredicted bands (4.8 kb; 2.8 kb; 2.3 kb) with the 3′-intron probe, suggesting that large indels likely occurred at the 3′ junctional integration site of the *ROSA*26 locus (Fig. 5B,C). Additionally, the B5 transgenic ferret also exhibited an additional band that hybridized to all three tested probes (a ~8 kb band using the 5′-intron probe, a ~6 kb and an over 12 kb band using the *EGFP* probe, and a ~8 kb band using 3′-intron probe), suggesting an additional random integration event. A specific banding pattern with expected sizes for a reverse orientation insertion (9.0 kb, 3.9 kb and 9.0 kb) of the transgene was observed in the genomic DNA of the B2 ferret using the internal *EGFP*, 5′-intron, and 3′-intron probes, respectively. This animal also has an unexpected 12 kb band detected by the *EGFP* probe, suggesting the occurrence of another random integration event (Fig. 5B,C).

### Ubiquitous expression of tdTomato in F1 transgenic ferrets harboring the *ROSA26*-targeted transgene cassette

**Figure 6:**
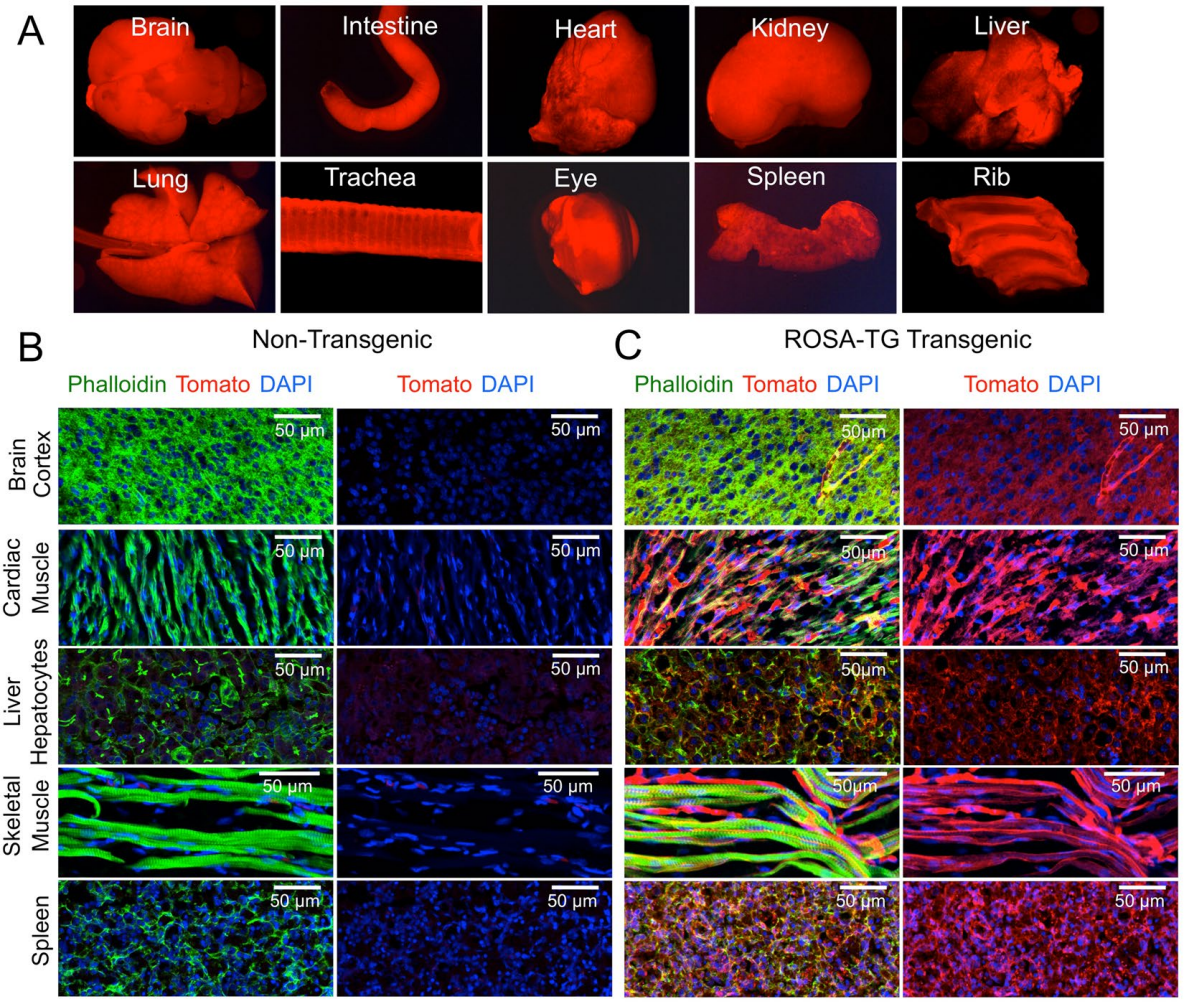
Global expression of the encoded *tdTomato* transgene in *ROSA26*-targeted (ROSA-TG) ferrets. (**A**) Representative *en face* fluorescent images of the indicated whole mount organs in the transgenic F1 newborn ferret (A1) demonstrate ubiquitous expression of the *tdTomato* transgene. (**B**,**C**) Fluorescent photomicrographs of phalloidin-stained (green) cryosections for brain cortex, cardiac muscle, liver hepatocytes, skeletal muscle and spleen from a newborn (**B**) non-transgenic and (**C**) transgenic ferret (A1). Phalloidin was used for staining filamentous actin (F-actin) to allow for better visualization of tissue structure. DAPI was used to stain nuclei.

**Figure 7:**
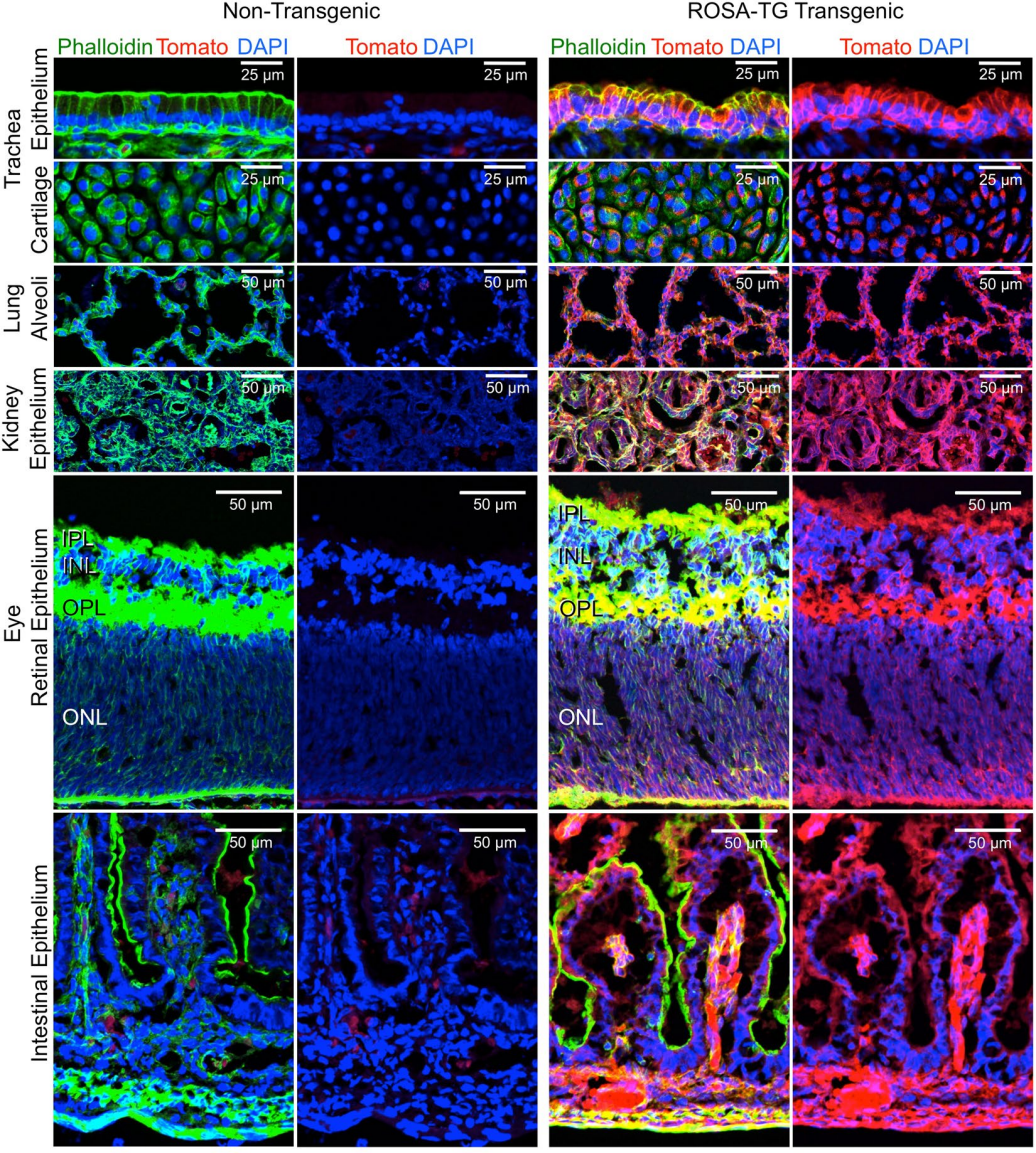
Global expression of the encoded *tdTomato* transgene in epithelial tissues of *ROSA26*-targeted (ROSA-TG) ferrets. Fluorescent photomicrographs of phalloidin-stained (green) cryosections of the indicated tissues including tracheal epithelium, tracheal cartilage, distal lung alveoli, kidney epithelium, retinal epithelium, and intestinal epithelium in a non-transgenic (left panels) and a transgenic F1 (A1; right panels) newborn ferret. DAPI was used to stain nuclei. INL, inner nuclear layer; IPL, inner plexiform layer; ONL, outer nuclear layer; OPL, outer plexiform layer. Phalloidin was used for staining filamentous actin (F-actin) to allow for better visualization of tissue structure. DAPI was used to stain nuclei.

To ensure that the *ROSA26* integration site enables global expression of its CAG promoter-driven transgene in all tissues, a F1 transgenic ferret from founder A1 was euthanized and dissected to assess tdTomato (mT) fluorescence in various organs. *En face* fluorescent images of brain, intestine, heart, kidney, liver, lung, trachea, eye, spleen and rib showed ubiquitous expression of mT in F1 offspring (Fig. 6A). Fluorescent images of sectioned organs further demonstrated broad expression of membrane-bound mT in brain cortex, cardiac muscle, liver hepatocytes, skeletal muscle and spleen (Fig. 6B). Ubiquitous expression of mT fluorescent protein was also observed in epithelial tissues, including tracheal epithelium and cartilage, distal lung alveoli, kidney epithelium, retinal epithelium and intestinal epithelium, but not in non-transgenic animals (Fig. 7). These results demonstrate that ferrets harboring a tdTomato-containing transgene cassette at the *ROSA26* locus exhibit a similar pattern of global expression as that observed in *Rosa26* knock-in mice harboring the identical transgene cassette^43^.

### Functional demonstration of Cre reporter activity of the ^LoxP^*tdTomato*^StopLoxP^*EGFP* cassette in transgenic ferret fibroblasts

**Figure 8:**
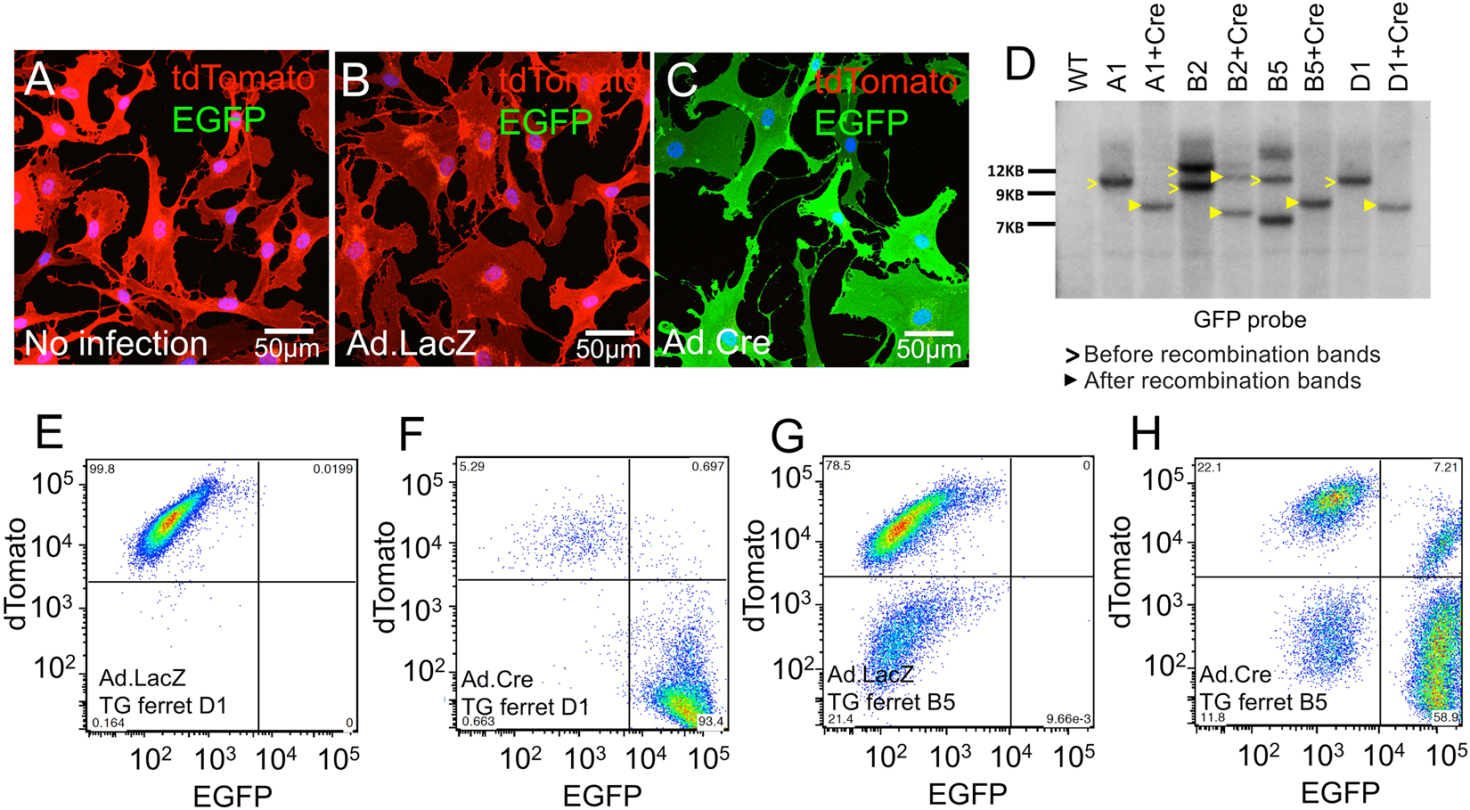
Functional analysis of Cre/LoxP-mediated recombination in fibroblast from *ROSA26*-targeted transgenic ferrets. Passage 4 (P4) primary fibroblasts derived from all surviving transgenic ferret founders were used for functional analysis of Cre-mediated recombination *in vitro*. (**A**–**C**) *In vitro* functional validation of Cre-mediated recombination in transgenic ferret fibroblasts (A1). Representative confocal fluorescent images for tdTomato and EGFP of the (**A**) mock control non-infected fibroblasts, (**B**) negative control Ad.LacZ-infected fibroblasts, and (**C**) experimental Ad.Cre-infected fibroblasts at 14 days post-infection. (**D**) Southern blotting analysis of Ad.Cre-infected transgenic ferret fibroblasts. Fibroblasts from the surviving A1, B2, B5 and D1 transgenic ferrets were infected with Ad.Cre and genomic DNA was extracted for Sac1-restricted Southern blotting against an *EGFP* probe at 14 days post-infection. Wild-type non-transgenic ferret DNA and non-infected fibroblasts of each transgenic animal served as negative controls for Cre-mediated excision of tdTomato. Open and closed arrowheads on the blot indicate the intact and tdTomato-deleted transgene fragment, respectively. (**E**–**H**) Representative FACS plots of tdTomato and EGFP fluorescence following Cre-mediated recombination in fibroblast cells from transgenic ferrets. Fibroblasts derived from transgenic ferret founders D1 (**E**,**F**) and B5 (**G**,**H**) were analyzed at 14 days after an infection of Ad.LacZ (**E**,**G**) and Ad.Cre (**F**–**H**). No EGFP conversion was detected by FACS in Ad.LacZ-infected fibroblasts derived from D1 (**E**) and B5 (**G**) animals, but striking conversions of tdTomato to EGFP were observed in cells infected with Ad.Cre from both transgenic founders D1 (**F**) and B5 (**H**). The two other transgenic founder lines (A1 and B2) demonstrated a FACS pattern following Ad.Cre infection similar to D1. Note that B5 fibroblasts has a subset of tdTomato negative cells in both Ad.Cre and Ad.LacZ infected cultures, suggesting this founder is a chimera with integration at the *ROSA26* locus occurring after division of the zygote. Values in the corners of the FACS plots indicate the percentage of cells in each quadrant.

We next sought to confirm the functionality of the Cre reporter within transgenic primary fibroblasts. Primary fibroblasts were derived from ear biopsies of transgenic ferrets and infected with a recombinant adenoviral vector expressing LacZ (Ad.LacZ; negative control) or Cre recombinase (Ad.Cre). As expected, there was efficient conversion of mT to membrane-bound EGFP (mG) following Ad.Cre infection in all the primary fibroblasts evaluated from live founders (Fig. 8A–C and data not shown). By contrast, uninfected fibroblasts (Fig. 8A) and Ad.LacZ-infected fibroblasts (Fig. 8B) retained the expression of mT with no conversion to mG. However, the B5 founder had a subset of mT negative cells in both the Ad.Cre and Ad.LacZ infected fibroblasts (Fig. 8G,H), suggesting that this founder was likely a chimera with most, but not all, fibroblasts containing the integrated *ROSA26* transgene. This B5 founder was the only transgenic founder that produced fibroblasts lacking mT expression. Southern blotting of genomic DNA from these fibroblasts using an *EGFP* probe further confirmed Cre-mediated excision of *dtTomat*o as indicated by a reduction in the size of the transgene cassette following Ad.Cre infection as compared to non-infected fibroblasts (Fig. 8D). FACS analysis of Ad.Cre-mediated recombination in fibroblasts from the four surviving transgenic ferret founders demonstrated highly efficient conversion of mT to mG (89.2% for A1, 96.1% for B2, 58.9% for B5, 93.5% for D1) (Fig. 8E–H, and data not shown). These results further support the fidelity of CRISPR/Cas9-mediated transgene integration events.

## Discussion

We have developed a protocol to efficiently generate targeted knock-in ferrets by building on previously described techniques that use a CRISPR/Cas9-mediated NHEJ integration approach and pronuclear injection^44,45^. By using this protocol, transgenic ferrets harboring ^LoxP^*tdTomato*^StopLoxP^*EGFP* dual reporter genes were generated using an sgRNA that concurrently cleaved both the ferret genomic *ROSA26* locus and the donor plasmid, allowing for targeted insertion without the need for long homology arms. In comparison to conventional homologous recombination^46^ and CRISPR/Cas9-mediated HDR approaches in mice^31,32^, results from Southern blotting analysis and germ-line transmission demonstrate that the CRISPR/Cas9-mediated NHEJ approach increased the efficiency of targeted integration in ferret zygotes. Despite this increased efficiency over HDR-based approaches, NHEJ-directed targeting integration has several drawbacks. First, although the approach is designed for directional insertion of the donor DNA, we observed that the insert effectively integrated in both the forward and reverse orientations. Second, indels at both the 3′- and 5′-junctions occurred, limiting the application for precisely introducing reporter constructs into genes. However, this can be circumvented using an intron-based insertion strategy that is compatible with capturing expression of a gene locus through the introduction of a splice acceptor within the transgene cassette.

The addition of phiC31/Bxb1 integrase sites within the transgene cassette should also allow for flexible and rapid insertion of a second transgene juxtaposed to the Cre reporter, or for the directional exchange of the Cre reporter for another transgene using the DICE system^39^. Such applications in *ROSA26*-CAG-^LoxP^tdTomato^StopLoxP^-EGFP (*ROSA*-TG) ferrets could utilize transgenic zygotes as the target, or alternatively, recombinant viruses to engineer somatic cells *in vivo*. In this regard, the DICE system was reported as a high-precision DNA integration tool at a desired genome location that can rapidly generate gain-of-function genetic models by direct embryo injection^39^. Furthermore, a DICE-mediated transgene exchange in 2~4-cell stage *ROSA*-TG zygotes would be predicted to generate chimeric animals that lose the fluorescent signal in those cells that gain expression of the exchanged transgene. Such an approach could be useful to study the cell autonomous function of genes.

In summary, we report a novel strategy for efficiently generating targeted transgene integration events in ferrets using a CRISPR/Cas9-mediated NHEJ approach. This transgenic ferret model containing a *ROSA*-TG dual-Cre reporter and integrase landing pad at the *ROSA26* locus provides a universal genetic background for engineering additional transgenic ferret models and enhances biomedical research that involves lineage tracing and the evaluation of gene and stem cell therapies.

## Materials and Methods

### Construction of the transgene cassette

To generate the transgene donor plasmid, the transgene cassette was cloned into pBlueScript SK II (+) (pBS-SKII) backbone plasmid (Agilent Technologies, La Jolla, CA) by multiple subcloning steps. In brief the following steps were involved: (1) A synthesized gBlock containing a linker of multiple restriction sites with *PhiC31 attP*, KpnI-*phiC31attP*-BamHI-NheI-XhoI-SpeI was first cloned into the KpnI/SpeI sites of pBS-SKII to generate an intermediate plasmid, pBS-SK-*Phi31attP*. (2) Another synthesized gBlock containing KpnI-BamHI-NheI-XhoI-pA-SphI-*Bxb1attP*-NotI was cloned into the KpnI/NotI sites of pBS-SKII to construct the intermediate plasmid, pBS-SK-pA-*Bxb1*. (3) The insert released from XhoI/NotI digestion of the pBS-SK-pA-*Bxb1* plasmid was subcloned into the XhoI/NotI sites of the pBS-SK-*Phi31attP* plasmid to generate an intermediate plasmid, pBS-SK-2*attP*s-pA. (4) A *ROSA*26-sgRNA (ccggcccgacttttggagaacatgcaccggtac) was then inserted to the upstream NgoMIV/KpnI sites of the pBS-SK-2*attP*s-pA plasmid to generate another intermediate plasmid, pSK-sgRNA-*Phi31attP*-*Bxb1attP*. (5) The pSK-sgRNA-*Phi31attP*-*Bxb1attP* plasmid was linearized by NotI/PciI restriction enzyme digestion and then a *ROSA*26-sgRNA (ggccgcccgacttttggagaacatgcacca) was subcloned into the sgRNA-*Phi31attP*-*Bxb1attP* cassette, resulting in the plasmid, pSK-sgRNA-*Phi31attP*-*Bxb1attP*-sgRNA. (6) The LoxP-mT-Terminator-LoxP-mG fragment, isolated from the *ROSA*26-mT/mG plasmid (Plasmid #17787, Addgene, Cambridge, MA) by PmeI/NheI digestion, was subcloned into the pSK-sgRNA-*Phi31attP*-*Bxb1attP*-sgRNA plasmid at the EcoRI/NheI sites to construct the final transgene donor plasmid, pSK-sgRNA-*Phi31attP*-mT/mG-*Bxb1attP*-sgRNA. All oligonucleotides were synthesized at Integrated DNA Technologies, Inc. (IDT, Coralville, IA). The transgene contains a CAG promoter/intron-driving expression of the LoxP*-tdTomato-*stop-LoxP*-EGFP* cassette as previously described^43^ and is flanked by *Bxb1attP* and *Phi31attP* sites.

### crRNA design and ribonucleoprotein complex preparation

crRNAs of the sgRNA were designed using CRISPOR (http://crispor.tefor.net) to target intron 1 of the *ROSA26* locus. Three sgRNAs were screened using *in vitro* Cas9 RNP cleavage assays against a PCR product of the target. The most efficient sgRNA was used for all studies. The sgRNA complex was first prepared by annealing the mixture containing equal molar amounts of crRNA and tracrRNA in IDT Duplex Buffer at a final concentration of 10 μM and slowly annealed at 95 °C, with stepwise cooling to room temperature at a rate of −5 °C/min. To prepare the active ribonucleoprotein (RNP) complex, the annealed sgRNA complex was then incubated with 1 μM of diluted Alt-R S.p. Cas9 Nuclease 3NLS (IDT, Coralville, IA) at a concentration of 1 μM in IDT Duplex Buffer for 30 min at room temperature. The crRNA and tracrRNA were also generated at Integrated DNA Technologies, Inc. (IDT, Coralville, IA).

### Animals and microinjection of zygotes

All animal experimentation was performed according to protocols approved by the Institutional Animal Care and Use Committees of the University of Iowa. Fertilized single-cell embryos (zygotes) were collected as previously described^42^. The mixture containing a final concentration of 200 ng/μl of RNP and 20 ng/μl transgene donor plasmid was microinjected (Femptojet, Eppendorf, Hamburg, Germany) into zygotes pronuclei (3~5 pl). Injected embryos were cultured in TCM-199 + 10% FCS medium overnight to the 2-cell stage prior to being transferred into primipara pseudopregnant jills^2,15^. The kits were naturally delivered in 42 days (full-term gestation). Non-transgenic siblings were used as controls.

### Analysis of transgene expression patterns in tissues

The tissue expression patterns of transgenic animals were evaluated in F1 newborn ferrets. Animals were euthanized by subcutaneously injecting pentobarbital sodium. Organs were first whole-mount imaged under a DMRB Fluorescent microscope (Leica, Wetzlar, Germany). Isolated organs were then post-fixed with 4% paraformaldehyde (PFA) in PBS at 4 °C for 72 hrs, and immersed in 50% optimum cutting temperature (OCT) compound (Sakura Tissue-Tek, Torrance, CA) in PBS at 4 °C for an additional 24 hrs prior to being embedded in OCT for cryosectioning (10 μm). For histologic visualization of transgene expression, cryosections were incubated in blocking butter (1% BSA in PBS, pH 7.4) for 30 min at room temperature, followed by incubation with Alexa Fluor™ 488 Phalloidin (Thermo Fisher, Waltham, MA, 1:500 diluted in blocking buffer) for 1 hr at 4 °C for F-actin labeling. After washing with PBS two times, the slides were mounted with ProLong™ Diamond Antifade Mountant with DAPI (Thermo Fisher) and imaged using a Zeiss 880 laser scanning confocal microscope (Carl Zeiss AG, Oberkochen, Germany).

### PCR genotyping and sequencing analyses of integration sites

Genomic tail DNA from newborn ferrets was extracted using the DNeasy Blood & Tissue Kit (Qiagen, Hilden, Germany). Transgene integrations were first evaluated by PCR using primer sets that amplify the targeted region of intron 1 of the *ROSA26* gene (wild type), and junctions for the anticipated forward and reverse integration using *ROSA26* junctional genomic primers and transgene-specific primers (Table 2 and schematic in Fig. 3A). The PCR amplicons were visualized on standard agarose gels and were confirmed by sequencing after gel extraction. The primer sequences, annealing temperatures, and expected sizes of PCR products for each primer set are listed in Table 2.

### Tissue culture, adenoviral infection, and FACS analysis

Ear biopsies of newborn transgenic ferrets were sterilely minced into small pieces and cultured in 10% FBS/DMEM, 5% CO_2_ at 37 °C on tissue culture plates. Fibroblasts at passage 4 (P4) were used for functional analysis or genomic DNA isolation for Southern blotting analysis. The P4 fibroblasts expressing mT were infected with recombinant adenovirus expressing Cre recombinase (Ad.CMV-Cre) or (Ad.CMV-LacZ) at a multiplicity of infection (MOI) of 100 particles/cell and were then cultured for an additional 14 days prior to analysis by fluorescent microscopy and FACS. The adenoviral vectors were generated by Viral Vector Core at the University of Iowa (Iowa City, IA, USA) (https://medicine.uiowa.edu/vectorcore).

### Southern blotting analysis

Southern blotting was used to characterize the genomic structure of integration sites and evaluate random integration events. Briefly, 15 μg of genomic DNA isolated from transgenic and non-transgenic fibroblasts was enzymatically digested using Sac I endonuclease. DNA fragments were resolved on a 1% agarose gel. The gel was rinsed twice in ddH_2_O and depurinated by incubating in 0.125 M HCl for 20 min. The DNA was denatured by incubating the gel in 0.5 M NaOH/1.5 M NaCl twice for 15 min before it was finally neutralized in 0.2 M Tris/2x SSC. The DNA then transferred to an Amersham Hybond-N+ membrane (GE Healthcare, Little Chalfont, United Kingdom) overnight and then UV crosslinked. The membranes were probed using standard methods and were randomly primed with P^32^-dCTP-labeled PCR templates against *EGFP* and two *ROSA26* intronic probes that flanked the integration site.

## Supplementary information

Figure S1
