# Supplementary material for: Highly Efficient Transgenesis in Ferrets Using CRISPR/Cas9-Mediated Homology-Independent Insertion at the ROSA26 Locus
Source: Sci Rep. 2019 Feb 13;9:1971. doi: 10.1038/s41598-018-37192-4 (PMC6374392; doi:10.1038/s41598-018-37192-4)
Supplement: Supplementary file 1 — Figure S1 [file 41598_2018_37192_MOESM1_ESM.pdf]

Highly Efficient Transgenesis in Ferrets Using CRISPR/Cas9-Mediated Homology-Independent Insertion at the *ROSA26* Locus

Miao Yu, Xingshen Sun, Scott R. Tyler, Bo Liang, Anthony M. Swatek, Thomas J. Lynch, Nan He, Feng Yuan, Zehua Feng, Pavana G. Rotti, Soon H. Choi, Weam Shahin[1] Xiaoming Liu, Ziying Yan and John F. Engelhardt

Correspondence to: john-engelhardt@uiowa.edu

This PDF file includes:

Figure. S1. Protein conservation between human, ferret and mouse for disease relevant pathways.

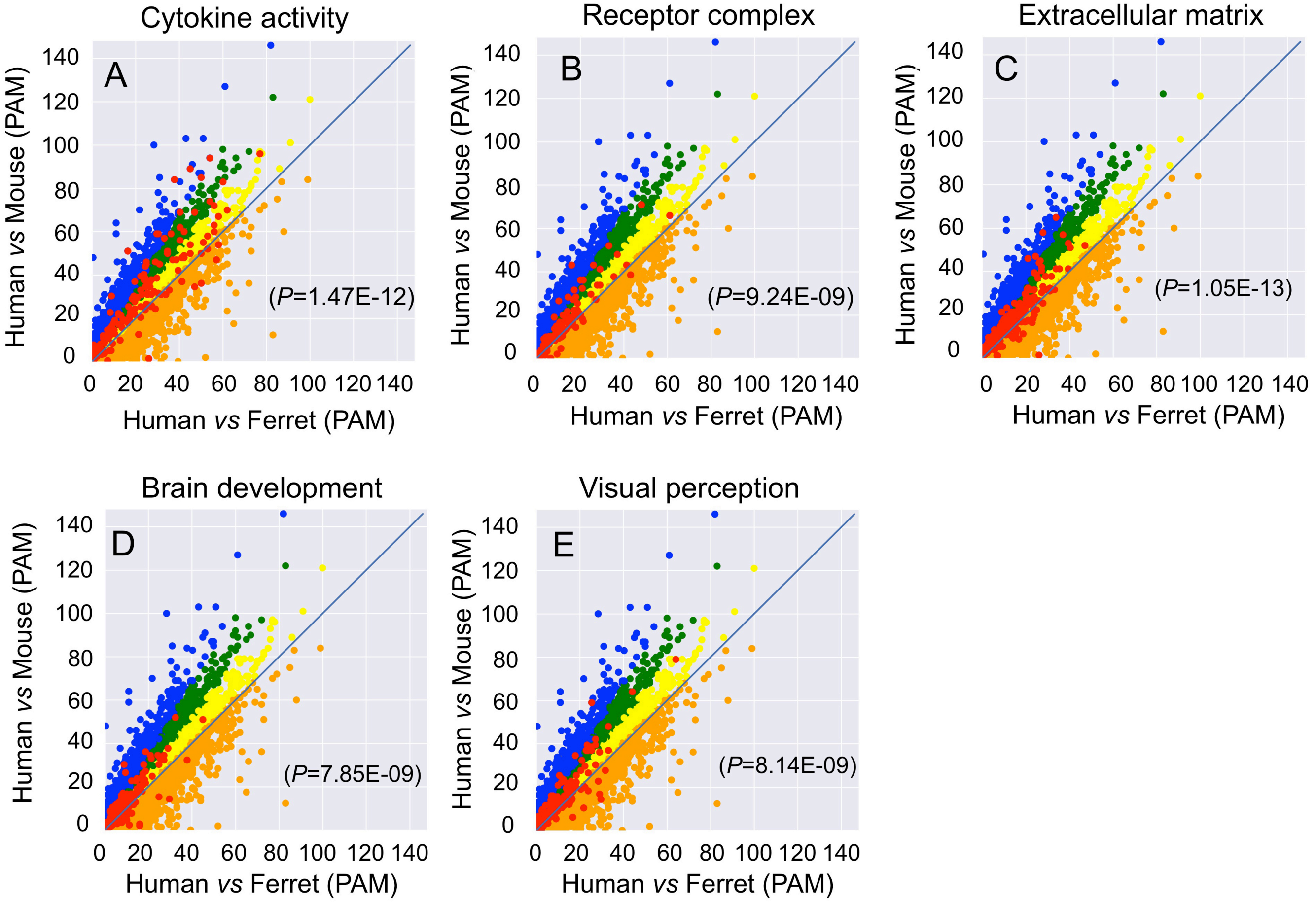

Figure S1. Protein conservation between human, ferret and mouse for disease relevant pathways. Proteome sequence conservation between human, ferret, and mouse plotted as the average protein sequence distance for three-way ortholog pairs. For each gene with mappable orthologs, the average distance between human and ferret, and the average distance between human and mouse, are displayed on the X- and Y-axes, respectively. Protein sequence distance was determined using a point-accepted mutation (PAM) substitution matrix [47]. Proteins for which the ferret and mouse sequences are equidistant from the human sequence fall on the blue line (slope=1). Proteins for which the conservation between ferret and human is better than that between mouse and human appear as dots above the blue line. Points below the blue line are better conserved between mouse and human. The angle of the line of each protein from the origin is directly related to the ratio of mouse divergence from human sequence and ferret divergence from human (mouse vs human/ferret vs human). A greater angle from the origin for any given point (i.e., protein) indicates a greater divergence in the mouse compared to the ferret; the quartiles of the distribution of these ratios comparing mouse and ferret divergence from human sequence are displayed in different colors (orange-brown being the least conserved in ferret relative to mouse, and blue being the most conserved in ferret relative to mouse). Proteins closer to the origin represent sequences that have diverged more slowly than proteins farther away from the origin. (**A-E**) GO-term subsets of proteins related to (A) cytokine activity, (B) receptor complex, (C) extracellular matrix, (D) brain development, and (E) visual perception were significantly better conserved (with human) in the ferret *vs*. mouse proteome (denoted by red dots), as determined by Wilcoxon Signed Rank test in R.

47. Dayhoff, M. O., Schwartz, R. & Orcutt, B. C. *A model of Evolutionary Change in Proteins*. (Silver Spring, 1978).
